# Zoonotic *Escherichia coli* and urinary tract infections in Southern California

**DOI:** 10.1128/mbio.01428-25

**Published:** 2025-10-23

**Authors:** Maliha Aziz, Daniel E. Park, Vanessa Quinlivan, Evangelos A. Dimopoulos, Yashan Wang, Edward H. Sung, Annie L. S. Roberts, Ann Nyaboe, Meghan F. Davis, Joan A. Casey, Julio Diaz Caballero, Keeve E. Nachman, Harpreet S. Takhar, David M. Aanensen, Julian Parkhill, Sara Y. Tartof, Cindy M. Liu, Lance B. Price, for the KP & GWU ARES team

**Affiliations:** 1 Department of Environmental and Occupational Health, George Washington University 8367, Washington, DC, USA; 2 Centre for Genomic Pathogen Surveillance Big Data Institute, Li Ka Shing Centre for Health Information and Discovery, University of Oxford 6396, Oxford, United Kingdom; 3 Department of Veterinary Medicine, University of Cambridge 2152, Cambridge, United Kingdom; 4 Department of Environmental Health and Engineering, Johns Hopkins School of Public Health 25802, Baltimore, Maryland, USA; 5 Department of Molecular and Comparative Pathobiology and Division of Infectious Diseases, Johns Hopkins School of Medicine 1500 , Baltimore, Maryland, USA; 6 Department of Research & Evaluation, Kaiser Permanente Southern California 82579 , Pasadena, California, USA; 7 Center for a Livable Future, Johns Hopkins University 1466, Baltimore, Maryland, USA; 8 Risk Sciences and Public Policy Institute, Johns Hopkins University 1466, Baltimore, Maryland, USA; 9 Department of Health Systems Science, Kaiser Permanente Bernard J. Tyson School of Medicine 547934, Pasadena, California, USA; 10 Department of Sequencing and Bioinformatics, Statens Serum Institut 4326, Copenhagen, Denmark; Iowa State University College of Veterinary Medicine, Ames, Iowa, USA

**Keywords:** zoonotic infections, urinary tract infection, genomic attribution model, extraintestinal pathogenic *E. coli*, *Escherichia coli*, antimicrobial resistance, Bayesian latent class model

## Abstract

Extraintestinal pathogenic *Escherichia coli* (ExPEC) is the leading cause of urinary tract infections (UTIs) worldwide and may be transmitted from food animals to humans via contaminated meat. However, the contribution of zoonotic ExPEC strains to UTIs in metropolitan areas remains unclear. We estimated the proportion of UTIs attributable to zoonotic ExPEC across eight Southern California counties. Between 2017 and 2021, we collected 12,616 *E. coli* isolates from retail meat and 23,483 from UTI patients, sequencing a representative subset of 5,728 isolates. Using a Bayesian latent class model trained with 17 host-associated genetic markers, we inferred the host origin of each isolate. Demographic, clinical, and antimicrobial resistance profiles were compared between meat isolates and clinical isolates inferred to be of human or food-animal origin. Most UTI patients were female (88%), with a median age of 50 years; 37% were Hispanic and 31% non-Hispanic white. Zoonotic ExPEC strains accounted for 18% of UTIs overall, rising to 21.5% in high-poverty neighborhoods. Women had a higher zoonotic proportion than men (19.7% vs 8.5%, *P* < 0.001). Among men, those with zoonotic infections were older than those with non-zoonotic infections (median 73.0 vs 65.0 years, *P* = 0.028). These findings underscore the contribution of zoonotic ExPEC to the UTI burden in Southern California and the need for targeted interventions to reduce risk in vulnerable communities.

**IMPORTANCE:**

Urinary tract infections (UTIs) are among the most common bacterial infections worldwide and are primarily caused by *Escherichia coli*. While *E. coli* is known to colonize both humans and food-producing animals, the extent to which zoonotic strains impact human disease remains poorly understood. Emerging evidence suggests that food animals may serve as an underrecognized reservoir for extraintestinal pathogenic *E. coli* (ExPEC). In this study, we used a genomic attribution model to quantify the contribution of zoonotic strains to UTIs in Southern California. We found that approximately 18% of *E. coli* UTIs were likely attributable to food animals. Individuals living in high-poverty neighborhoods had a 1.6-fold increased risk of zoonotic UTIs compared to those in low-poverty areas. These findings highlight zoonotic transmission as an important driver of UTIs and suggest that reducing ExPEC in food-animal reservoirs could help lower disease burden and address health disparities.

## INTRODUCTION

*Escherichia coli* causes a wide range of infections, including cystitis, pyelonephritis, meningitis, and sepsis, accounting for approximately one million deaths worldwide each year (1, 2). Extraintestinal pathogenic *E. coli* (ExPEC) strains, which cause infections outside of the gastrointestinal tract (3–5), are major contributors to the 8 million urinary tract infections (UTIs) in the United States each year (6, 7). While UTIs are typically considered low-acuity infections, they are responsible for substantial loss in productivity and medical costs (8) and can progress to serious invasive infections. More than half of *E. coli* sepsis cases originate as UTIs, making the urinary tract a major gateway to the bloodstream (9, 10). A better understanding of the ecology and epidemiology of ExPEC strains could inform novel strategies to reduce their disease burden.

Previous studies suggest that food animals may serve as reservoirs for zoonotic ExPEC strains (11). However, differentiating zoonotic strains from strains endemic to humans is challenging due to the sporadic nature of most extraintestinal *E. coli* infections and the vast census of food-producing animals—in the United States alone, more than nine billion animals are raised for slaughter annually (12).

Core-genome phylogenetic analysis is the gold standard for tracking the source of infectious diseases, but it is most effective for outbreak investigations involving distinct strains emanating from a single point source or a single introductory event. This approach relies on phylogenetic placement and relatedness thresholds; however, it is ineffective for resolving the sources of sporadic extraintestinal *E. coli* infections because it would require broad sampling of the potential source populations, but this is not feasible due to the enormous human and food-animal populations and the ubiquity of *E. coli*. Despite this limitation, genomic sequencing can still yield actionable insights by shifting the focus from identifying a single point source (e.g., a slaughterhouse) to identifying a primary host species (e.g., broiler chicken vs human).

In 2023, we described a novel statistical-genomic model for inferring the host origin of *E. coli* strains based on both core-genome phylogeny and mobile genetic elements (MGEs) differentially associated with food animals and humans (13). In the current study, we applied this Bayesian latent class model to a large contemporaneously collected set of *E. coli* isolates from retail meat and humans with UTIs in Southern California. Our objective was to estimate the proportion of UTIs caused by zoonotic ExPEC strains and to identify demographic and socioeconomic factors associated with increased infection risk. These findings will help inform targeted public health strategies to mitigate the burden of zoonotic UTIs in vulnerable populations.

## RESULTS

### Isolate collection and genomic analysis

We analyzed 5,728 *E. coli* genomes, including 2,349 human clinical isolates from positive urine cultures and 3,379 isolates from retail meat samples (chicken *n* = 970, turkey *n* = 792, pork *n* = 837, beef *n* = 780) purchased concurrently in the same residential areas as the clinical isolates between February 2017 and May 2021. Most clinical isolates were from female patients (88%), with a median age of 50 years (Q1–Q3: 31–68) (Fig. S1). The patients were predominantly Hispanic (37%) or non-Hispanic white (31%) (Table 1). The majority of patients resided in areas with low and medium family poverty rates (42.2% and 44.5%, respectively) (Table 1). Among the meat samples, *E. coli* contamination was highest among turkey (82%), followed by chicken (58%), pork (54%), and beef (47%). *E. coli* contamination was higher from meat samples purchased in areas with higher poverty rates—with a 12% increase in contamination with each 10% absolute increase in regional family poverty rate (aOR = 1.12, *P* = 0.018)—and higher among samples from value packs (aOR = 1.18, *P* = 0.003).

**TABLE 1:** Demographics and clinical outcomes of patients with zoonotic vs non-zoonotic infections

Parameter	A. All clinical (*n* = 2,349)	B. Zoonotic clinical (*n* = 416)	C. Non-zoonotic clinical (*n* = 1,933)	*P* value B vs C
Sex, *n* (col %)				
Female	1,723 (87.6)	334 (94.6)	1,389 (86.1)	<0.001
Male	244 (12.4)	19 (5.4)	225 (13.9)	<0.001
Age, median (Q1–Q3)	53.0 (33.0–69.0)	53.0 (35.0–71.0)	53.0 (33.0–69.0)	
Female	50.0 (31.0–68.0)	52.0 (34.0–70.0)	49.0 (30.0–67.0)	
Male	65.5 (54.0–75.5)	73.0 (61.0–80.0)	65.0 (54.0–75.0)	
Body mass index, median (Q1–Q3)	27.1 (23.2–31.5)	27.5 (23.2–31.8)	27.0 (23.3–31.4)	
Female	26.7 (23.0–31.2)	27.5 (23.1–31.9)	26.6 (23.0–31.0)	
Male	28.6 (25.3–33.0)	29.4 (25.7–31.8)	28.5 (25.3–33.0)	
Race and ethnicity, *n* (col %)				
Asian	147 (6.3)	27 (6.5)	120 (6.2)	0.838
Black	120 (5.1)	16 (3.9)	104 (5.4)	0.194
Hispanic	862 (36.7)	157 (37.7)	705 (36.5)	0.651
White	738 (31.4)	138 (33.2)	600 (31.0)	0.360
Other	482 (20.5)	78 (18.8)	404 (20.9)	0.316
Socio-economic data^*a*^				
Family poverty rate				0.071
Low (<8%)	984 (42.2)	67 (16.3)	821 (42.7)	
Medium (8%–14%)	1,038 (44.5)	182 (44.2)	856 (44.6)	
High (>14%)	311 (13.3)	163 (39.6)	244 (12.7)	
Encounter class, *n* (col %)				
Ambulatory	1,322 (56.3)	228 (54.8)	1,094 (56.6)	0.531
Virtual	540 (23.0)	112 (26.9)	428 (22.1)	0.038
Other	487 (20.7)	76 (18.3)	411 (21.3)	0.165

^*a*^ By three-digit ZIP code tabulation area.

The clinical and meat *E. coli* isolates comprised diverse sequence types, with substantially more unique sequence types (STs) identified among meat *E. coli* isolates than clinical isolates. We identified 306 unique STs among clinical isolates and 604 unique STs among meat isolates (chicken *E. coli* STs = 209, turkey = 185, pork = 192, beef = 275). The STs showed clear distinctions: clinical isolates were predominantly ST131 (11.6%), ST73 (11.4%), ST95 (9.9%), ST69 (9.4%), and ST127 (9.2%), while meat isolates were dominated by ST117 (5.4%), ST10 (4.1%), ST101 (3.9%), ST399 (3.6%), and ST58 (2.7%).

### Proportion of clinical urine *E. coli* isolates attributable to zoonotic ExPEC strains

**Fig 1:**
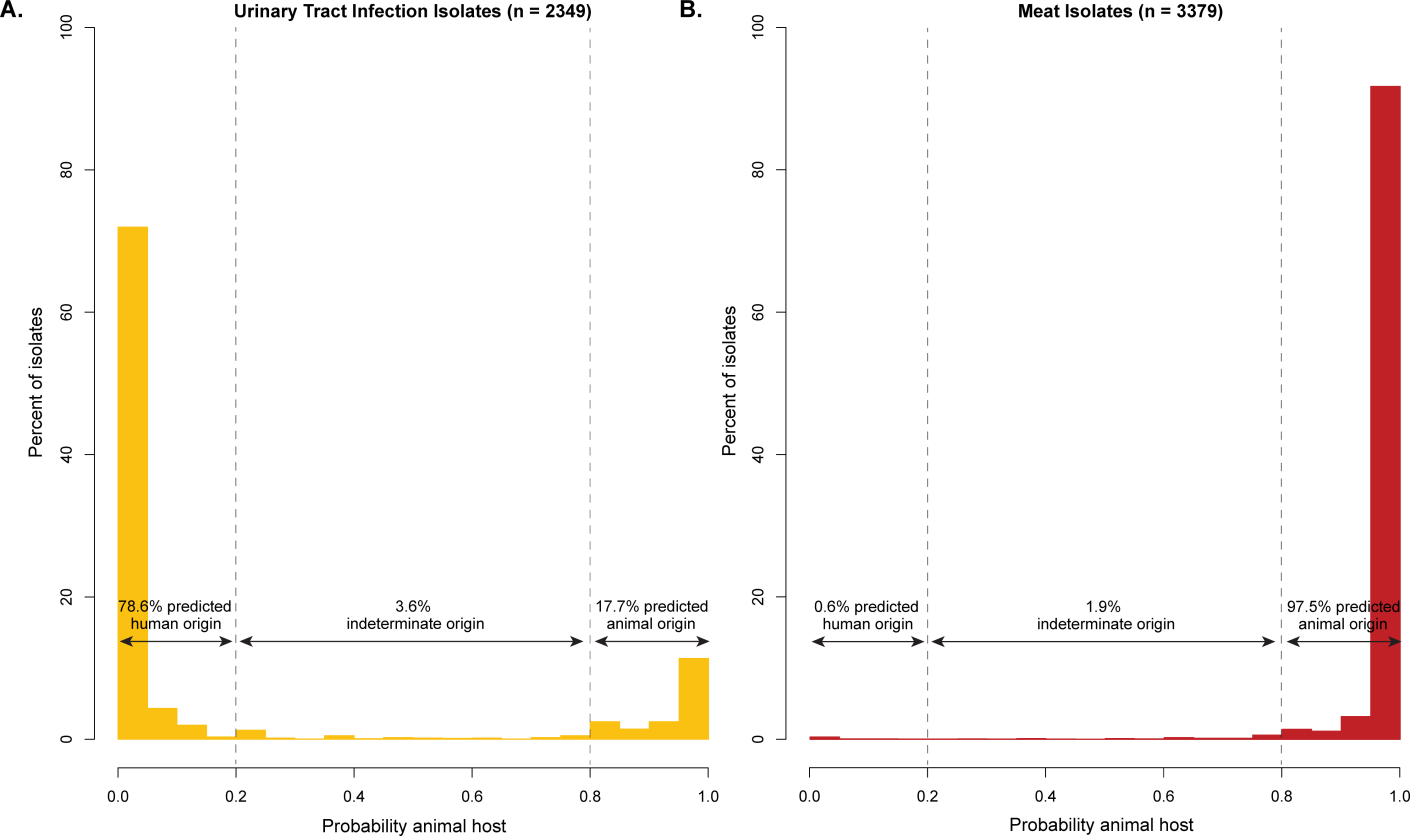
Host origin predictions for *E. coli* isolates from urinary tract infections and meat. The probability that isolates were derived from a food-animal host was estimated using a Bayesian latent class model based on core-genome mutations and 17 host-associated mobile genetic elements. (**A**) Probabilities for urinary tract infection isolates. Isolates with probabilities ≥0.8 (right of the dashed line at 0.8) were categorized as zoonotic ExPEC. (**B**) Probabilities for meat isolates.

**Fig 2:**
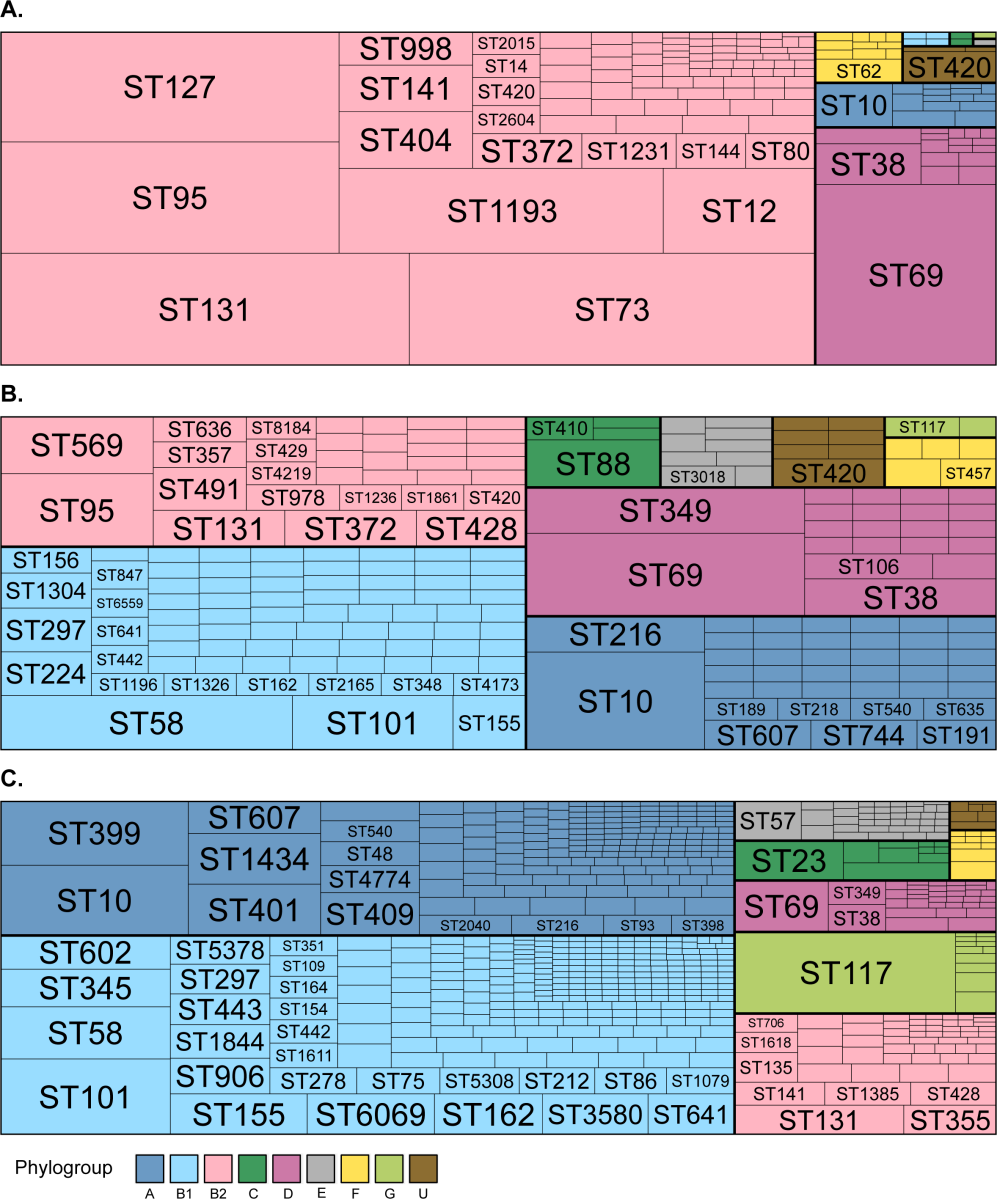
Tree maps of *E. coli* phylogroups and sequence types. (**A**) Non-zoonotic urinary tract infection isolates; (**B**) zoonotic urinary tract infection isolates; and (**C**) meat isolates. Rare sequence types are represented by a scaled box but non-individually named. The full list of sequence types can be found in Table S3.

Overall, the prevalence patterns of MGEs indicate distinct source-specific distributions, with several MGEs showing differential enrichment between human and meat-associated *E. coli* isolates. Notably, elements such as M1, M2, M4, and M5 were highly prevalent in chicken and turkey sources, whereas human-associated elements (e.g., H2, H3, H6) were more commonly detected in isolates from urinary tract infections (Table S1). Using our Bayesian latent class model (BLCM), we estimated that 17.7% of the human clinical urine *E. coli* isolates in our Southern California study population originated from food animals, classifying them as zoonotic ExPEC strains (Table S2; Fig. 1A). From this point forward in the manuscript, we use the term “zoonotic ExPEC” to refer to isolates inferred to be of food-animal origin based on our Bayesian latent class model. In contrast, only 0.6% of the meat isolates were inferred to be of human origin (Table S2; Fig. 1B). Most clinical and meat *E. coli* isolates received highly probable (>95%) source inference scores, with only a small fraction being of indeterminate origin (<80% source inference score) (clinical: 3.6%; meat: 1.9%). Zoonotic ExPEC strains were distributed amongst 196 sequence types; many (13.0% of STs) were shared across non-zoonotic ExPEC, zoonotic ExPEC, and meat isolates; however, the distribution of sequence types was distinct among these *E. coli* groups (Table S2; Fig. 2). Non-zoonotic ExPEC isolates were predominantly clustered in the B2 and D phylogroups, which are strongly associated with extraintestinal pathogenesis (14, 15), while the meat isolates were primarily of B1 and A phylogroups. Zoonotic ExPEC isolates had a more balanced distribution across A, B1, B2, and D phylogroups compared to the other two *E. coli* groups.

### Antimicrobial resistance patterns

Antimicrobial resistance gene profiles were significantly different between zoonotic ExPECs, non-zoonotic ExPECs, and meat isolates (pairwise permutational multivariate analysis of variance [PERMANOVA] *P* values all *P* < 0.001). Principal coordinate analysis (PCoA) of the antimicrobial resistance genes revealed distinct clustering among the clinical isolates and among the meat isolates (Fig. S2). Zoonotic ExPEC isolates exhibited intermediate profiles, overlapping partially with both clinical and meat groups.

Multidrug resistance was significantly more prevalent among non-zoonotic ExPEC isolates as compared to zoonotic ExPEC isolates (Table S4); however, no significant difference in resistance was observed between zoonotic ExPEC and meat isolates. Meat and zoonotic ExPEC isolates tended to show lower resistance to clinically relevant classes of antimicrobials, except for tetracyclines. Gentamicin resistance was significantly higher in meat isolates than both zoonotic and non-zoonotic clinical isolates.

### High-risk zoonotic ExPEC lineages linked to human urinary infections

Zoonotic ExPEC-associated sequence types were most common in isolates from retail chicken (38%) and turkey (36%), followed by beef (14%) and pork (12%). A small subset of *E. coli* lineage from meat was disproportionately responsible for a large portion of the ExPEC strains identified in human clinical urine samples, suggesting that these virulent animal-associated strains represent the greatest zoonotic risk (Fig. S3). The B2 and D phylogroups were prevalent among clinical isolates and rare among meat isolates; the A and B1 phylogroups showed the opposite trend. Notable sequence types included ST69, which belongs to the D phylogroup and was the most prevalent among the zoonotic ExPEC strains, and ST117, which belongs to the G phylogroup and was abundant in meat but rarely observed among zoonotic ExPEC isolates. ST10, ST58, and ST101, which belong to the less virulent A and B1 phylogroups, were also prevalent among the zoonotic ExPEC isolates. Extraintestinal virulence genes were more commonly found in ST10, ST58, and ST101 isolates compared to other A and B1 sequence types (PERMANOVA, *P* < 0.001) (Fig. S4) (Table S5).

### Zoonotic ExPEC strains disproportionately affect women and older men

Urine samples from women were significantly more likely to have zoonotic ExPEC strains than samples from men (19.7% vs 8.5%, *P* < 0.001) (Table 1). Among men, those with zoonotic ExPEC strains were significantly older than those with non-zoonotic ExPEC strains (median 73.0 years vs 65.0 years, *P* = 0.028), while a similar, though less pronounced, age difference was also observed in women (median 52.0 years vs 49.0 years, *P* = 0.027). No significant differences were found between zoonotic and non-zoonotic patient populations in terms of body mass index, race/ethnicity, or type of clinical visit.

### Socioeconomic disparities in zoonotic ExPEC infections

**Fig 3:**
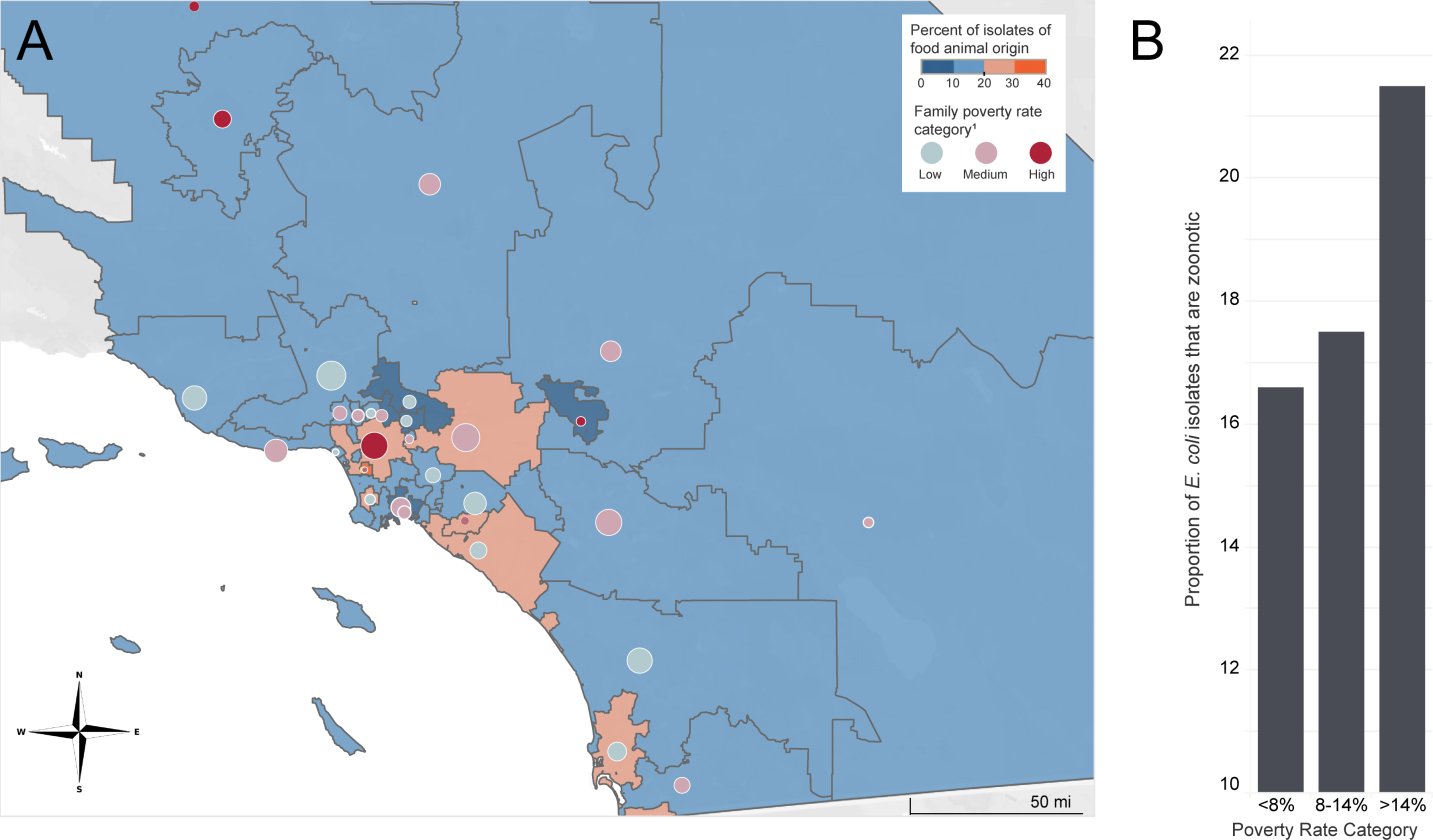
Association between family poverty rate and zoonotic urinary tract infection rates in Southern California. (**A**) Geospatial distributions of zoonotic urinary tract infections in each three-digit ZIP code tabulation area overlaying the family poverty rate category. The size of each circle is proportional to the sample size of urinary tract infection isolates from each respective region, ranging from 7 to 277. (**B**) Dose response between poverty rate category and proportion of urinary tract infections inferred to be zoonotic.

In addition to host demographic differences, we also evaluated the potential contribution of community socioeconomic status to zoonotic ExPEC infection risk. The highest proportion of zoonotic ExPEC isolates was identified among patients residing in areas with high poverty rates (21.5% of all clinical *E. coli* isolates), while lower proportions of zoonotic ExPEC isolates were found in areas with medium (17.5%) and low (16.6%) poverty rates. After adjusting for age, sex, and race/ethnicity, individuals residing in a high-poverty area had a 1.6-fold increased risk of zoonotic ExPEC infections compared to those in low-poverty areas (*P* = 0.008). Spatial patterns further supported these findings, showing consistent geographic correlations between poverty levels and zoonotic ExPEC transmission (Fig. 3).

## DISCUSSION

Zoonotic ExPEC strains appeared to be responsible for nearly 18% of UTIs among beneficiaries of Kaiser Permanente Southern California from 2017 to 2021. Our analysis also suggested that those living in the poorest communities may be at the greatest risk for these infections. The factors underlying the strong correlation between poverty rate and zoonotic UTIs are unclear. Possible explanations may include contamination of meat products marketed in resource-poor communities due to inadequate adherence to food safety regulations, suboptimal retail conditions (e.g., improper storage temperatures), deficiencies in food handling and hygiene practices (16), and duration prior to sale (17). Additionally, behavioral factors related to food preparation and consumption, particularly in settings with limited water, sanitation, and hygiene, may further exacerbate contamination risks (18). These putative factors could work synergistically to drive up zoonotic infection rates in poorer communities. Other factors unrelated to consumption, such as occupational exposure to meat, could be important determinants. Socioeconomic inequities have been implicated in higher prevalence of UTIs in this patient population (17); therefore, zoonotic transmission may represent a small but significant risk factor among a network of other determinants of UTI risk.

The proportion of zoonotic infections estimated in this Southern California population was approximately twofold higher than what we estimated from a similar study in Flagstaff, Arizona (13). The reason for this difference is unclear. The two populations differed in size, location, socio-economic status, and demographic makeup, with the Southern California region having greater socio-economic inequities and heterogeneity compared with the Flagstaff region. The studies were also conducted almost 10 years apart; therefore, multiple factors may have contributed to the different proportions.

The overall proportion of antimicrobial resistance was comparable between the current Southern California study and the prior Arizona study for both clinical *E. coli* isolates (52.9% vs 54.9%) and meat isolates (64.7% vs 67.5%). However, resistance to ampicillin and tetracycline—both targeted by California Senate Bill 27—was lower in the current study among meat isolates (20.5% vs 33.0% and 34.4% vs 49.9%, respectively) and clinical isolates (42.4% vs 45.1% and 17.1% vs 24.7%). Although relative prescribing rates for the Arizona study were not available, penicillins were among the most commonly dispensed antibiotics for uncomplicated *E. coli* UTIs in the Kaiser Permanente Southern California network (19). Thus, the lower resistance observed is unlikely to be attributable to reduced clinical prescribing alone and may reflect changes in agricultural use. Notably, zoonotic UTIs in the current study had resistance patterns similar to those of meat isolates. Together, findings suggest that reductions in antimicrobial resistance among food animals may lead to clinically meaningful decreases in resistance among zoonotic UTIs.

A subset of *E. coli* lineages, primarily from poultry products, appeared to have enhanced capacity to cause zoonotic UTIs. The prevalence of *E. coli* sequence types among zoonotic UTIs was not significantly correlated with their prevalence among meat isolates, suggesting that the capacity to cause infections was primarily driven by inherent virulence rather than abundance in the food supply. A substantial body of work suggests that the B2 and D phylogroups have the greatest capacity to cause extraintestinal infections (14, 15, 20–23). Here, sequence types belonging to these phylogroups were prevalent among infection isolates despite being relatively rare among meat isolates. ST10, ST58, and ST69 also demonstrated enhanced capacity to cause UTIs despite belonging to the A and B1 phylogroups. Further research is required to determine the specific factors underlying the increased zoonotic potential of these three sequence types. The highest risk zoonotic ExPEC strains were most prevalent among poultry *E. coli* populations. Contamination rates were also highest among poultry products, especially turkey meat, suggesting that these products may pose the greatest infection risk.

Overall, the zoonotic ExPEC strains were significantly less likely than the non-zoonotic ExPEC strains to be resistant to antibiotics important for treating UTIs (i.e., sulfamethoxazole and trimethoprim; Table S4) (19), with resistance patterns shifting in the direction of those observed in meat isolates. Since the early 1990s, the United States Food and Drug Administration’s Center for Veterinary Medicine has implemented several important rules and guidance documents meant to limit the use of medically important antimicrobials in food animals (24–26). Our results, especially when compared to data from countries where regulations are more lax (27), may support the effectiveness of these efforts to reduce the threat of antimicrobial-resistant pathogens from food animals.

Strengths of the study included a statistical-genomic approach to infer the host origins of *E. coli* isolates and a large data set with contemporaneously collected samples from a region that includes both metropolitan and rural areas with heterogeneous socioeconomic conditions. Although relatively novel, this statistical-genomic approach is becoming increasingly utilized as a complementary tool to traditional epidemiologic and source attribution approaches (13, 28, 29). Limitations of the study included our inability to infer specific meat types with the current version of the model (V.1.1.0) (30). Furthermore, the model was not developed with *E. coli* genomes from cattle or beef products, which could have resulted in an underestimation of the contribution of cattle-origin isolates to human disease. We expect that future iterations of the model will overcome these limitations by including additional host-associated MGEs. Additionally, we could not differentiate between foodborne exposure and other exposure pathways. We anticipate that a small proportion of *E. coli* isolates may derive from recurring UTIs (31); we expect that this would bias the zoonotic proportion toward the null due to both MGE loss and observed susceptibility patterns. Furthermore, this study focused on community-acquired infections by targeting outpatient visits. Hospital- and healthcare-acquired infections are likely subject to different environmental pressures and zoonotic contributions (32). Finally, because our study did not include strains from invasive infections, we may have been limited in our ability to identify the zoonotic ExPEC strains that may pose the greatest public health risk. However, given that most invasive *E. coli* infections begin in the urinary tract (10), the study likely captured the major strains that could cause ascending UTIs, including urosepsis (5, 33).

In conclusion, nearly one out of five UTIs in Southern California may be caused by zoonotic ExPEC strains. Recognizing this route of exposure presents new opportunities for reducing the heavy burden of UTIs. Our findings underscore the need for interventions to reduce the prevalence of zoonotic ExPEC in the food supply. Meat producers and regulatory agencies should consider more stringent measures to limit the prevalence of ExPEC in retail meat, particularly poultry, which appears to be a significant reservoir. Future research should further investigate whether policies designed to lower antimicrobial resistance in food animals can drive meaningful reductions in antimicrobial resistance in human infections. Enhanced surveillance, stricter processing controls, and targeted interventions along the supply chain will be critical to mitigating the risk of transmission to humans. Until such measures are widely adopted, individuals at heightened risk of bacterial infections—including immunocompromised individuals, older adults, and those with chronic conditions—should exercise caution when handling raw meat (including frozen). Preventive steps, such as thorough handwashing, avoiding cross-contamination, and ensuring proper cooking of meat products, are essential to reducing exposure. Addressing this overlooked pathway of ExPEC transmission is imperative for protecting public health and reducing the incidence of foodborne UTIs.

## MATERIALS AND METHODS

### Study design—human clinical *E. coli* collection

This cross-sectional analysis has been previously described (34). Briefly, we collected human clinical *E. coli* isolates from positive urine culture at the Kaiser Permanente Southern California regional labs on a weekly basis from February 2017 to May 2021, with preferential sampling from ambulatory care visits as determined by encounter class designations extracted from the electronic health record. The laboratory served Kaiser Permanente Southern California (KPSC) patients who were residents from Bakersfield to San Diego (Fig. S5). KPSC is an integrated healthcare system with over 4.8 million members across 16 hospitals and 226 clinics across the Southern California region. The highly diverse racial/ethnic and sociodemographic distribution reflects that of the surrounding region (35, 36).

Urine *E. coli* isolates were recovered in the clinical laboratory using standard techniques. Briefly, urine specimens were collected by KPSC and processed for routine clinical purposes from 2016 to 2021. Agar plates of isolated *E. coli* were shipped to GWU for further testing.

### Study design—meat *E. coli* collection

Concurrently, retail meat samples were also collected weekly in the same catchment area as the human clinical *E. coli* collection, as described (34). Briefly, raw chicken, turkey, pork, and beef samples were collected weekly from all major grocery chains in Southern California. A single *E. coli* isolate was recovered from each culture-positive meat product using enrichment methods as described previously (37). Antimicrobial susceptibility for the meat isolates was determined by disk diffusion in accordance with the Clinical and Laboratory Standards Institute (38). Antibiotics included in susceptibility testing for both clinical and meat isolates included ampicillin, ciprofloxacin, gentamicin, tetracycline, and trimethoprim-sulfamethoxazole. Meat isolates were also tested for amoxicillin clavulanic acid, azithromycin, cefoxitin, chloramphenicol, colistin, doxycycline, kanamycin, and streptomycin. Temporal and geographic patterns of susceptibility among chicken *E. coli* isolates have been described previously (37).

### Data collection

De-identified data were extracted from the electronic health records systems at KPSC for clinical isolates, including sex, age, date of isolate collection, and residence three-digit ZIP code. For retail meat samples, we collected information, including date of collection, brand, USDA establishment code, grocery or retail location, organic or conventional production, raised without antibiotics labeling, and meat processor.

### Human clinical and meat *E. coli* selection for whole-genome sequencing

From 2017 to 2021, we collected 23,483 human clinical urine *E. coli* isolates and 12,616 retail meat samples from across southern California (Fig. S6). Most patients were female (86%) and self-reported being Hispanic (43%) or White (37%) (Table S6). From the retail meat and poultry samples, we cultured 6,313 *E. coli* isolates from chicken, 2,768 from turkey, 1,361 from pork, and 2,540 from beef. Contamination calculations exclude data from July 2018 to June 2019; during that time period, the lab protocol was changed to include freezing of retail meat samples prior to isolate recovery, reducing isolate viability during this period only.

To generate a representative subset of *E. coli* isolates for whole-genome sequencing, we randomly subsampled all *E. coli* isolates based on calendar year, animal or human origin, and week of collection. Specifically, we designed the subsample strategy to select 6,000 total *E. coli* isolates with an even distribution of isolates across each calendar year (2017–2021), an equal number of human and animal isolates per year, and a target of at least 15 isolates per week. Due to disruptions in sampling due to the COVID-19 pandemic in 2020, we selected all available meat isolates from 2020 and continued sampling into 2021.

A total of 7,563 human clinical and meat *E. coli* isolates were selected for sequencing. The subsampled human clinical *E. coli* collection was generally representative of the total patient population (Table 1).

### Sequencing and genomic analysis

DNA was isolated from clinical and meat specimens using PureLink Pro 96 Genomic DNA Purification Kit (Invitrogen) and sequenced on the Illumina NovaSeq platform at Sanger Institute, UK. Illumina adapters were removed using AdapterRemoval (39) (v2.3.2), and short reads were passed through read length and base quality checks using FastQC (40) (v0.11.9). Reads that passed a phred score ≥30 and read length ≥15 bp were retained. To check for *E. coli–E. coli* contamination, isolate reads were mapped to a reference genome using Bowtie2 aligner (41) and single-nucleotide polymorphisms (SNPs) were identified using GATK4 (42). Any genome with heterozygous SNPs *N* > 1,250 (median + 2.5 × median absolute deviation) was removed (43, 44). Isolate reads that passed the quality filters were assembled into contigs using SPAdes (45) (v3.15.3), and only genomes <6.14 MB were retained. Assemblies were annotated using bakta (46) (v1.5.1). Sequence types were assigned to isolate genomes using mlst (47) (v2.19.0); phylogroup assignment was performed using EzClermont (48); antimicrobial resistance determinants were identified using ResFinder (49) (v4.4.2) with cutoff values of 80% nucleotide identity and 80% query coverage. Resistance patterns were compared based on lab-based phenotypic susceptibility testing and *in silico* predictions using ResFinder (Table S7). Assembled genomes were screened against the Extended-ExPEC virulence panel using VirulenceFinder (50) (database version 2.0.0) with cutoff values of 80% nucleotide identity and 80% query coverage.

### Model inputs

We applied our previously established in-house pipeline (30), which defines core-genome clusters and screens for MGEs to generate model inputs. As detailed in reference 13, raw Illumina reads from human, chicken, turkey, and pork-derived *E. coli* isolates are assembled and annotated with Prokka v1.14.5 (51), then used to build a pangenome via Roary v3.12.0 (52). Accessory genes showing significant source-specific associations are identified through pairwise statistical comparisons, and highly correlated loci (Pearson > 0.7) are clustered into MGEs after expert curation (Table S8). We extended this framework by replacing traditional core-genome phylogenetic clades with core‐genome multilocus sequence typing (cgMLST) clusters. Briefly, cgMLST was assigned to a globally curated *E. coli* data set using the EnteroBase Escherichia cgMLST scheme (53). An in-house k-modes clustering model (30) was trained on cgMLST profiles, and the model was used to assign cgMLST-based clusters to new *E. coli* genomes. An in-house script from the pipeline was used to detect the presence/absence of host-associated MGEs using minimap2 (54) (v2.24) (-cx asm20). An MGE was called present if any one gene from the element was present in the genome. The resulting clusters were appended to the MGE presence/absence data for further analysis using the BLCM.

### Inferring host origin using a Bayesian latent class model

We inferred the host origin of each isolate based on a combination of genotype and the presence/absence of 17 host-associated MGE (13) using a Bayesian latent class model (55). The Bayesian latent class analysis is uniquely able to incorporate differential sensitivity and specificity patterns from the panel of MGEs to generate probabilistic predictions of host origin for individual isolates. The model assumes there are human or meat classes and uses patterns of CGE clustering and MGEs to generate a probability of being in a human or meat class for each isolate. Response probabilities were generated using independent logistic normal priors with a mean of 0 and a standard deviation of 1.5; for the class probabilities (human vs meat), a Beta (1, 1) prior was used.

### Statistical analysis

Demographic, clinical, and antimicrobial susceptibility characteristics were compared between meat isolates, zoonotic human isolates, and human-origin isolates using chi-squared, Fisher’s exact, and Wilcoxon rank sum tests, as appropriate. Differences in resistance determinants and virulence genes across groups were assessed using PCoA and PERMANOVA, and differences in variance were assessed using beta dispersion analysis. A multivariate logistic regression model was fit to assess the association between *E. coli* contamination and predictors, including family poverty rate (scaled per 10% increase), production type (organic/raised without antibiotics and conventional), meat type, value-pack status, and adjusted for year of sample collection.

Associations between zoonotic transmission and socioeconomic factors (median household income and family poverty rate) were conducted, with spatial analyses conducted in Tableau. Median household income by three-digit ZIP code tabulation area (ZCTA) was extracted from the 2020 United States Census Bureau Decennial Census, while the family poverty rate by three-digit ZCTA was calculated from the 2020 United States Census Bureau American Community Survey. Median household income was not used in the final analyses as there was a minimum household income with minimal variance, potentially due to subsidies or other programs. Family poverty rate was defined as the percent of households living below the federal poverty threshold, categorized into three groups: low (<8%), medium (8%–14%), and high (>14%) based on clustering patterns on histogram distributions. Associations between family poverty rate categories and zoonotic transmission were assessed using a binomial generalized linear model, adjusted for sex, race/ethnicity, age, and medical encounter type.

We excluded missing data from statistical comparisons. Analyses were conducted in SAS (SAS Institute Inc., v.9.4, Cary, NC, USA), Tableau (2023.3.2), R (v.3.3.1), and with Bayesian inference software JAGS v.4.2.0.

## Data Availability

Short read sequencing data for the *E. coli* isolates included in this study are available on NCBI, SRA accession number PRJEB31347. Anonymized patient data that support the findings of this study may be made available from the investigative team in the following conditions: (i) agreement to collaborate with the study team on all publications, (ii) provision of external funding for administrative and investigator time necessary for this collaboration, (iii) demonstration that the external investigative team is qualified and has documented evidence of training for human subjects protections, and (iv) agreement to abide by the terms outlined in data use agreements between institutions. All code developed for this analysis is available at (i) https://github.com/antonisdim/5708, (ii) https://github.com/cgps-group/sb27_analyses, and (iii) https://github.com/araclab/general/tree/main/Food-epidemiology/host_element.

## SUPPLEMENTAL MATERIAL

Supplemental material mbio.01428-25-s0001.pdf Supplemental figures and tables.

Supplemental tables mbio.01428-25-s0002.xlsx Tables S2 and S3.

ASM does not own the copyrights to Supplemental Material that may be linked to, or accessed through, an article. The authors have granted ASM a non-exclusive, world-wide license to publish the Supplemental Material files. Please contact the corresponding author directly for reuse.
