# Supplementary material for: Zoonotic Escherichia coli and urinary tract infections in Southern California
Source: mBio. 2025 Oct 23;16(11):e01428-25. doi: 10.1128/mbio.01428-25 (PMC12607791; doi:10.1128/mbio.01428-25)
Supplement: Supplemental material — Supplemental figures and tables. [file mbio.01428-25-s0001.pdf]

Supplemental Tables and Figures

Zoonotic *Escherichia coli* and Urinary Tract Infections in Southern California

Contents

Fig S1. Histogram of age in years of UTI patients, stratified by gender...2

Table S1. Prevalence of the 17 source-associated mobile genetic elements in each sample type.............3

Fig S2. PcoA plot of resistance genes among E. coli isolates from meat and from zoonotic and non-zoonotic E. coli isolates from clinical urine specimens...4

Table S4. *In silico* predicted antimicrobial susceptibility of isolates from meat, zoonotic infections, and non-zoonotic infections. 5

Fig S3. Prevalence of sequence types among E. coli isolates from meat vs zoonotic urinary tract infections..6

Fig S4. PcoA plot for extended extra-intestinal pathogenic virulence genes found from ST10, ST58, and ST101 versus all other sequence types from phylogroups A and B1...7

Table S5. Virulence factors significantly different between ST10, ST58, ST101 (Group1) and STs from other B1 and A phylogroups (Group2)..8

Fig S5. Approximate catchment area for the Kaiser Permanente Southern California Network.................9

Fig S6. Flowchart of isolate selection and sequencing. 10

Table S6. Comparing total clinical *E. coli* isolate collection to final sequenced clinical *E. coli* isolate collection...11

Table S7. Antimicrobial susceptibility for isolates from meat, zoonotic infections, non-zoonotic infections comparing phenotypic susceptibility to in silico predicted (ResFinder) susceptibility..............................12

Table S8. Characteristics of the 17 source-associated mobile genetic elements (MGEs) identified in (1), including MGE type, number of source-associated accessory genes per element, source association, known features and putative selective functions...14

Tables S2 and S3 are provided in a separate supplemental Excel file

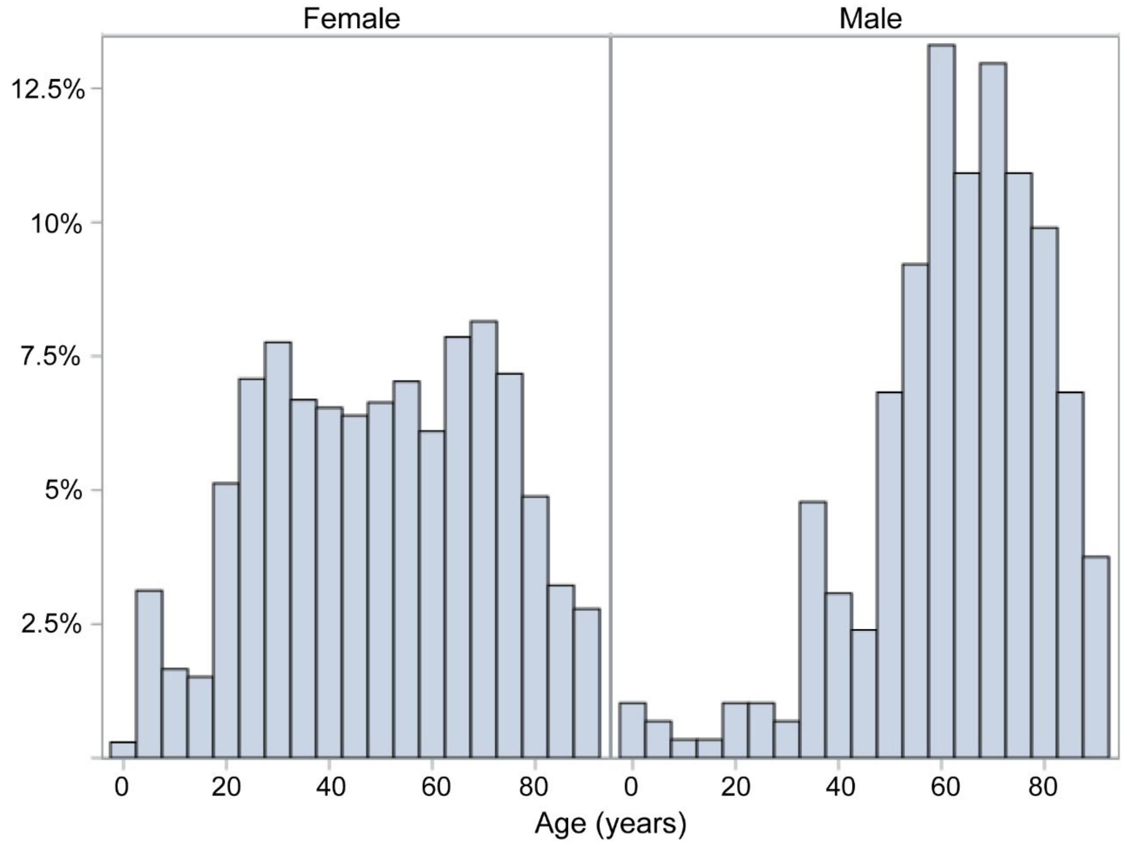

Fig S1. Histogram of age in years of UTI patients, stratified by gender.
*Ages >89 are reported as 89 years of age.

Table S1. Prevalence of the 17 source-associated mobile genetic elements in each sample type.

Element	Human n=2350	Chicken n=970	Turkey n=792	Pork n=837	Beef n=780
H1	33.74	5.57	13.01	2.03	4.62
H2	45.91	2.78	1.14	3.23	2.69
H3	59.91	3.40	8.21	2.99	2.69
H4	16.09	0.00	0.00	0.00	0.64
H5	14.00	2.47	22.85	3.35	1.67
H6	62.51	0.62	1.01	0.96	0.77
M1	31.19	59.48	42.05	52.93	40.26
M2	28.81	45.15	35.61	30.11	45.51
M3	8.77	41.86	54.04	7.05	6.15
M4	15.02	63.61	63.64	35.84	37.56
M5	8.47	76.49	88.51	25.45	11.03
M6	1.96	27.63	52.15	13.98	3.97
M7	9.74	20.41	9.60	5.73	2.82
M8	2.21	30.62	50.38	54.72	18.21
M9	1.15	15.15	21.34	5.73	1.41
M10	2.30	19.07	32.07	13.02	4.87
M11	2.55	19.69	35.73	47.55	16.41

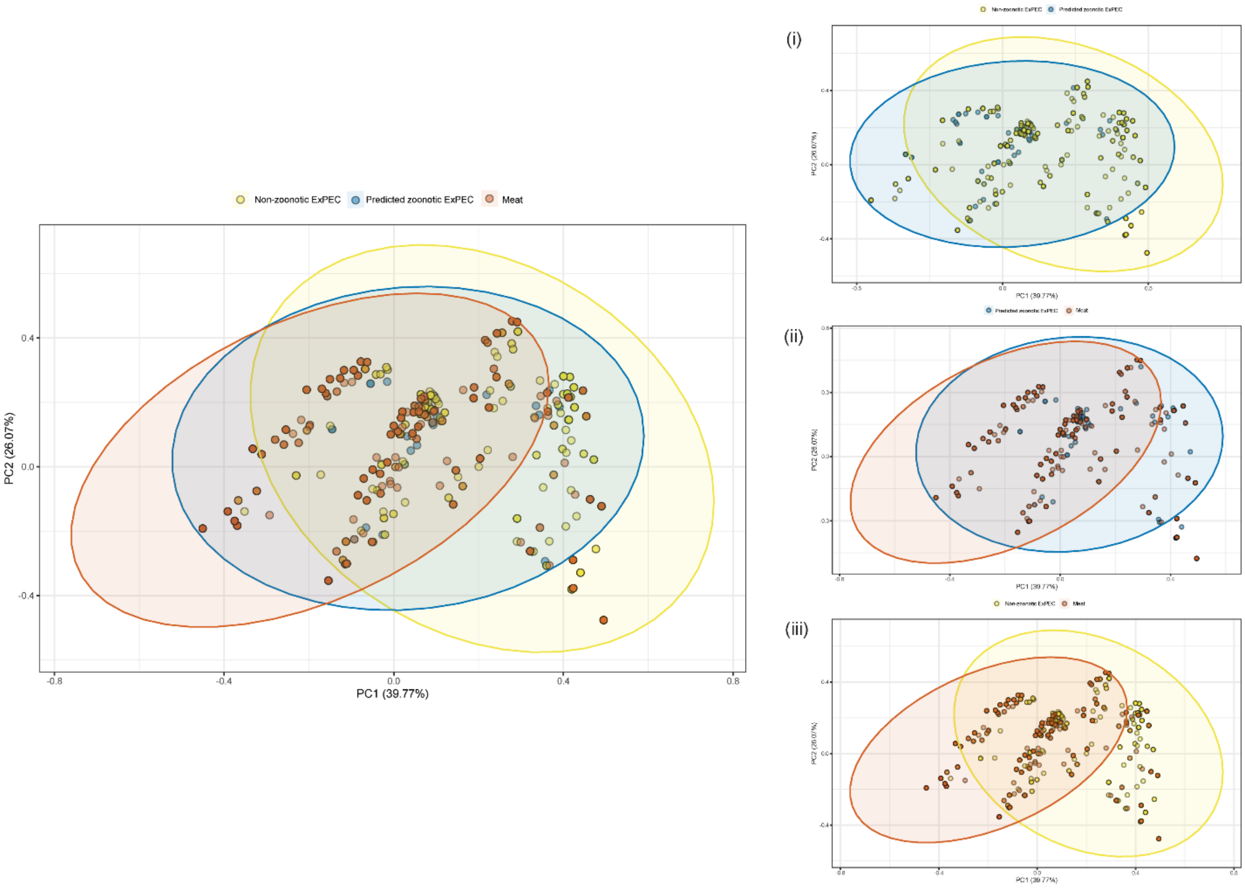

Fig S2. PcoA plot of resistance genes among E. coli isolates from meat and from zoonotic and non-zoonotic E. coli isolates from clinical urine specimens.

Blue circles, zoonotic isolates; yellow circles, non-zoonotic isolates; red circles, meat isolates; large ovals denote confidence ellipses for the three groups and captures the variation in resistance gene profiles within each group; i) non-zoonotic and zoonotic *E. coli* isolates from clinical urine specimens (PERMANOVA R^2=1.75% p<0.001); ii) zoonotic *E. coli* from clinical urinary tract infections and meat (PERMANOVA R^2=2.32% p<0.001); iii) non-zoonotic *E. coli* from clinical urine specimens and meat (PERMANOVA R^2=1.29% p<0.001).

Table S4. ***In silico*** **predicted antimicrobial susceptibility of isolates from meat, zoonotic infections, and non-zoonotic infections.**

	**Meat Isolates**	**Human Clinical Isolates (n=2349)**			
	**A. Meat origin (n=2559)**	**B. predicted zoonotic (n=416)**	**C. non-zoonotic (n=1933)**	**Chi-square p-value**	
	**% Isolates (n)**			**A vs B**	**B vs C**
**Resistance to Antimicrobial Classes**					
0	54.8 (1402/2559)	60.1 (250/416)	49.2 (950/1933)	0.043	<0.001
≥ 1	45.2 (1157/2559)	39.9 (166/416)	50.9 (983/1933)	<0.001	<0.001
≥ 2	30.1 (769/2559)	30.5 (127/416)	38.4 (743/1933)	0.163	0.002
≥ 3 (multidrug resistant)	17.4 (444/2559)	22.8 (95/416)	31.3 (604/1933)	0.137	<0.001
**Resistance to Individual Antimicrobials**					
Amoxicillin Clavulanic Acid (β-Lactam/β-Lactamase Inhibitor)[1]	1.3 (34/2559)	2.4 (10/416)	2.0 (38/1933)	0.093	0.567
Ampicillin (β-lactam)[1]	13.2 (337/2559)	26.0 (108/416)	41.9 (810/1933)	<0.001	<0.001
Azithromycin (Macrolide)	0.2 (6/2559)	2.9 (12/416)	15.7 (304/1933)	<0.001	<0.001
Cefazolin (Cephalosporin)[1]	N/A	N/A	N/A	N/A	N/A
Ceftazidime (Cephalosporin)[1]	N/A	N/A	N/A	N/A	N/A
Cefoxitin (Cephalosporin)	1.1 (29/2559)	1.7 (7/416)	0.3 (5/1933)	0.345	<0.001
Ceftriaxone (Cephalosporin)[1]	N/A	N/A	N/A	N/A	N/A
Chloramphenicol (Amphenicol)	2.0 (51/2559)	5.5 (23/416)	2.6 (51/1933)	<0.001	0.002
Ciprofloxacin (Fluoroquinolone)[1]	0.7 (18/2559)	9.9 (41/416)	4.1 (79/1933)	<0.001	<0.001
Colistin (Polymyxin)	0.0 (0/2559)	0.2 (1/416)	0.0 (0/1933)	0.013	0.031
Doxycycline (Tetracycline)	36.5 (933/2559)	27.6 (115/416)	23.9 (461/1933)	<0.001	0.103
Gentamicin (Aminoglycoside)	11.7 (300/2559)	3.9 (16/416)	7.5 (145/1933)	<0.001	0.007
Kanamycin (Aminoglycoside)	8.0 (204/2559)	4.6 (19/416)	1.0 (20/1933)	0.014	<0.001
Nitrofurantoin[1]	N/A	N/A	N/A	N/A	N/A
Piperacillin-Tazobactam (β-Lactam/β-Lactamase Inhibitor)[1]	1.3 (34/2559)	2.4 (10/416)	2.0 (38/1933)	0.093	0.567
Streptomycin (Aminoglycoside)	24.0 (615/2559)	22.8 (95/416)	31.2 (603/1933)	0.578	<0.001
Sulfamethoxazole (Sulfonamide)[1]	19.4 (497/2559)	21.4 (89/416)	31.5 (609/1933)	0.360	<0.001
Tetracycline (Tetracycline)	36.5 (933/2559)	27.6 (115/416)	23.9 (461/1933)	<0.001	0.103
Trimethoprim (Antifolate)[1]	3.5 (89/2559)	19.0 (79/416)	25.4 (491/1933)	<0.001	0.006

[1] In a class of antibiotics commonly used to treat uncomplicated *E. coli* urinary tract infections in the KPSC network, based on Jennifer H Ku, Katia J Bruxvoort, S Bianca Salas, et al. Multidrug Resistance of Escherichia coli From Outpatient Uncomplicated Urinary Tract Infections in a Large United States Integrated Healthcare Organization, *Open Forum Infectious Diseases*, Volume 10, Issue 7, July 2023, ofad287, https://doi.org/10.1093/ofid/ofad287

*N/A (Not Applicable): The antimicrobial drugs were tested in the lab but are not listed in the ResFinder database.

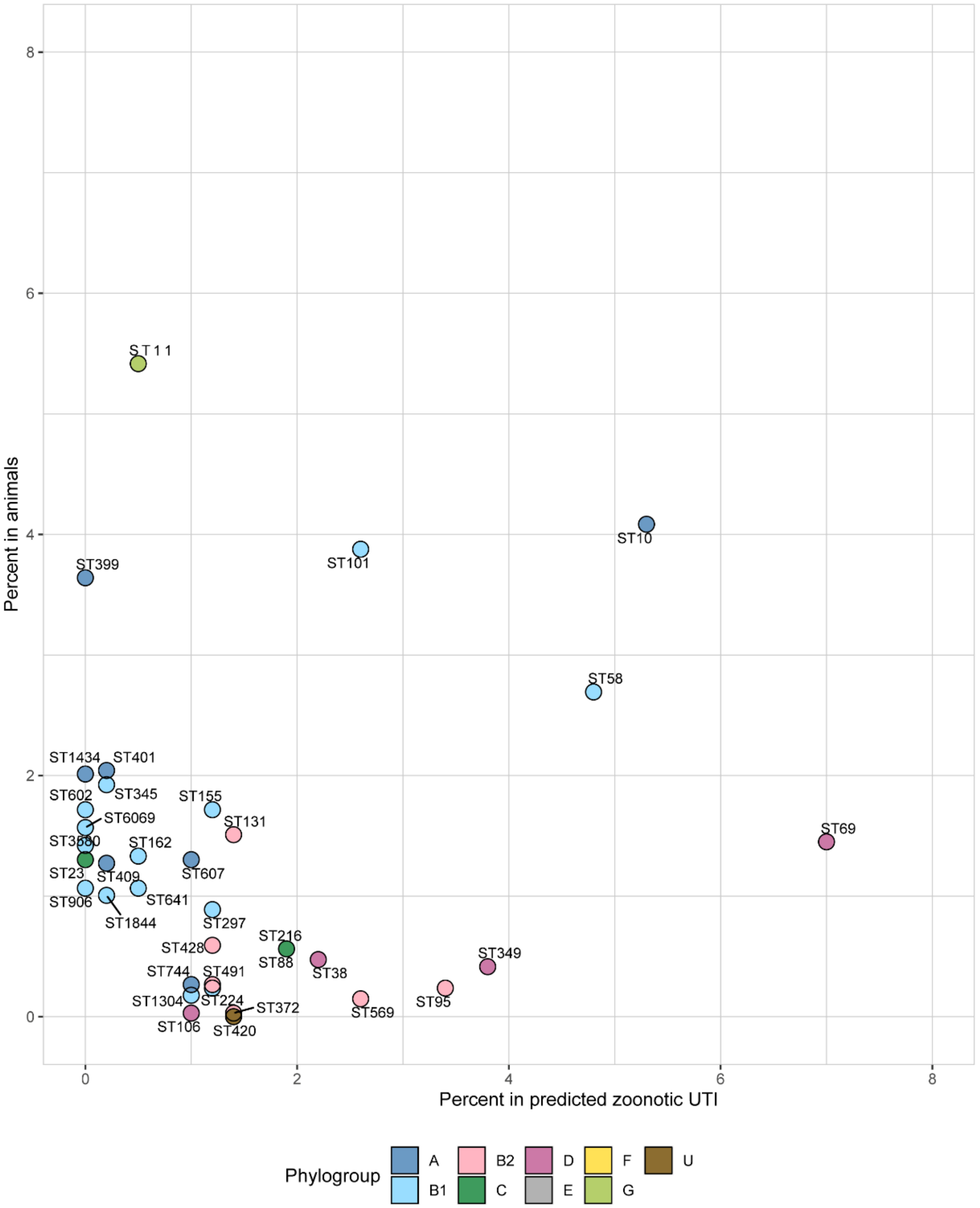

Fig S3. Prevalence of sequence types among E. coli isolates from meat vs zoonotic urinary tract infections.

Prevalence of MLSTs among zoonotic ExPEC isolates plotted against their prevalence among meat isolates. The circles are colored by phylogroup the MLSTs belong to. Only sequence types observed in >=1% of isolates from either meat or UTIs are shown.

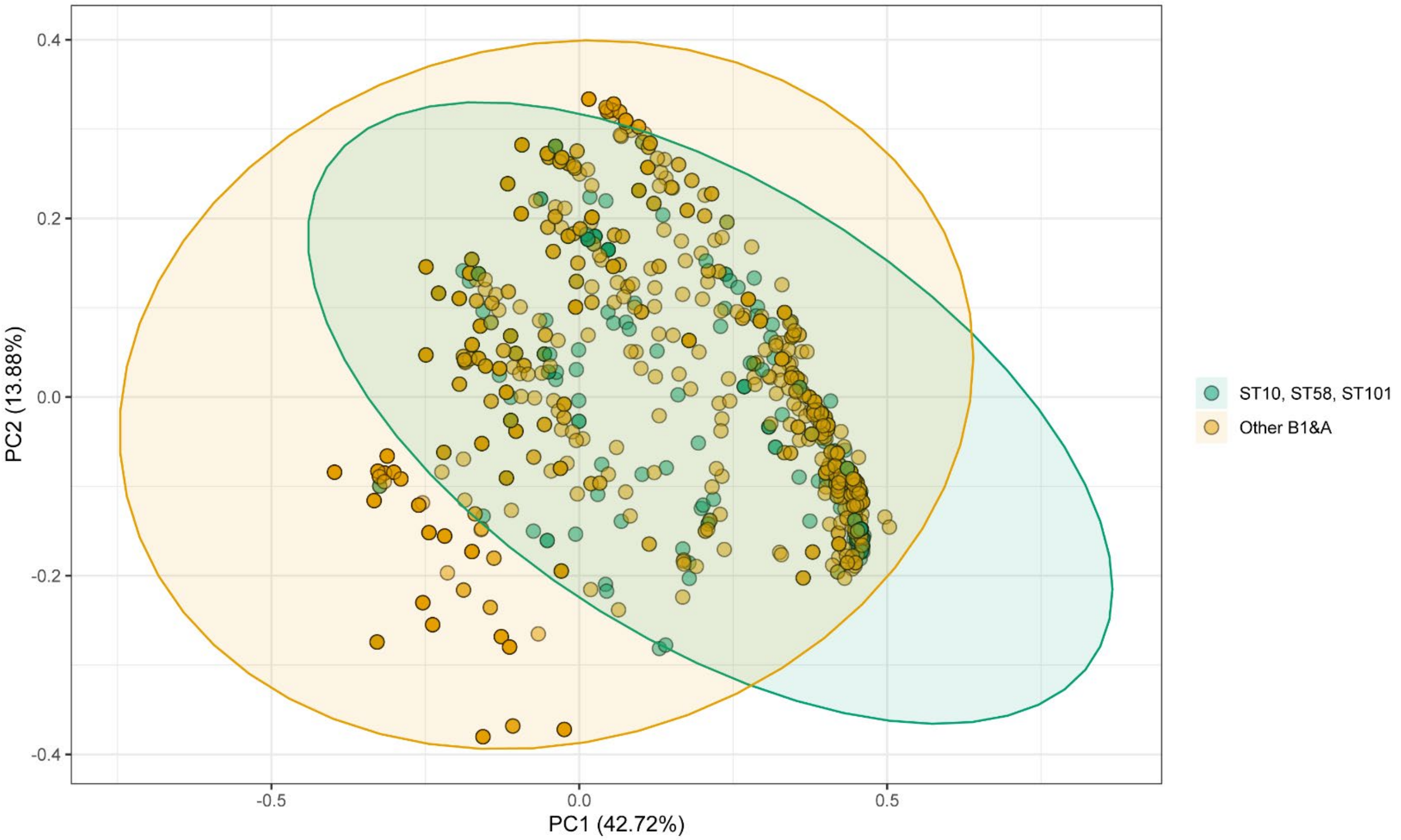

Fig S4. PcoA plot for extended extra-intestinal pathogenic virulence genes found from ST10, ST58, and ST101 versus all other sequence types from phylogroups A and B1.

Blue circles represent ST10, ST58, and ST101 isolates; orange circles represent other A and B1 isolates; large ovals denote confidence ellipses for the two groups and captures the variation in virulence gene profiles within each group types (PerMANOVA, R^2=0.026, p<0.001).

Table S5. Virulence factors significantly different between ST10, ST58, ST101 (Group1) and STs from other B1 and A phylogroups (Group2)

Gene	Prevalence		p-value	Gene detail	Description
	Group1	Group2			
afaA	8.3	2.4	<0.001	Transcriptional regulator	Afimbrial adhesin
afaB	48.6	25.3	<0.001	Periplasmic chaperone	Afimbrial adhesin
afaC	80.6	55.1	<0.001	Outer membrane usher protein	Afimbrial adhesin
afaD	3.9	0.6	<0.001	Afimbrial adhesion	Afimbrial adhesin
afaE	3.9	0.4	<0.001	Adhesin protein	Afimbrial adhesin
cea	3.7	0.5	<0.001	Colicin E1	transmembrane toxins
cia	1.8	0.5	0.0073	Colicin Ia	transmembrane toxins
cib	8.8	5.7	0.0215	Colicin Ib	transmembrane toxins
cvaC	11.8	6.6	<0.001	Microcin C	ColV operon (Colonization)
chuA	4.8	1.2	<0.001	Outer membrane hemin receptor	Iron uptake
cnf1	23.3	11.8	<0.001	Cytotoxic necrotizing factor 1	Toxin
focC	1.4	0.1	<0.001	S fimbrial/F1C minor subunit	F1 fimbriae (adhesin)
sfaE/sfafoCDE	43.3	19.9	<0.001	S fimbrial/F1C minor subunit	S fimbriae (adhesin)
sfaD	1.4	0.3	0.0041	S fimbrial/F1C minor subunit	S fimbriae (adhesin)
focG	44.0	31.1	<0.001	F1C adhesion	F1C fimbriae (adhesin)
hra	40.3	8.9	<0.001	Heat-resistant agglutinin	Coding for Heat-resistant agglutinin
ibeA	99.8	90.0	<0.001	Invasin of brain endothelial cells	Invasin
ireA	47.2	25.2	<0.001	Siderophore receptor	Iron uptake
iroN	17.1	9.4	<0.001	Enterobactin siderophore receptor protein	salmochelin operon (Iron uptake)
iucC	43.3	16.9	<0.001	Aerobactin synthetase	Aerobactin operon (Iron uptake)
iutA	43.3	16.9	<0.001	Ferric aerobactin receptor	Aerobactin operon (Iron uptake)
kpsE	5.8	3.1	0.0103	Capsule polysaccharide export inner membrane protein	Capsule polysaccharide transport
ompT	67.3	47.1	<0.001	Outer membrane protease (protein protease 7)	Protease
papA	9.9	3.0	<0.001	Major pilin subunit	P fimbriae (Adhesins)
sitA	51.6	33.2	<0.001	Iron transport protein	Iron uptake
iss	39.9	8.8	<0.001	Increased serum survival	serum resistance factor genes
traT	65.2	47.0	<0.001	Outer membrane protein complement resistance	serum resistance factor genes

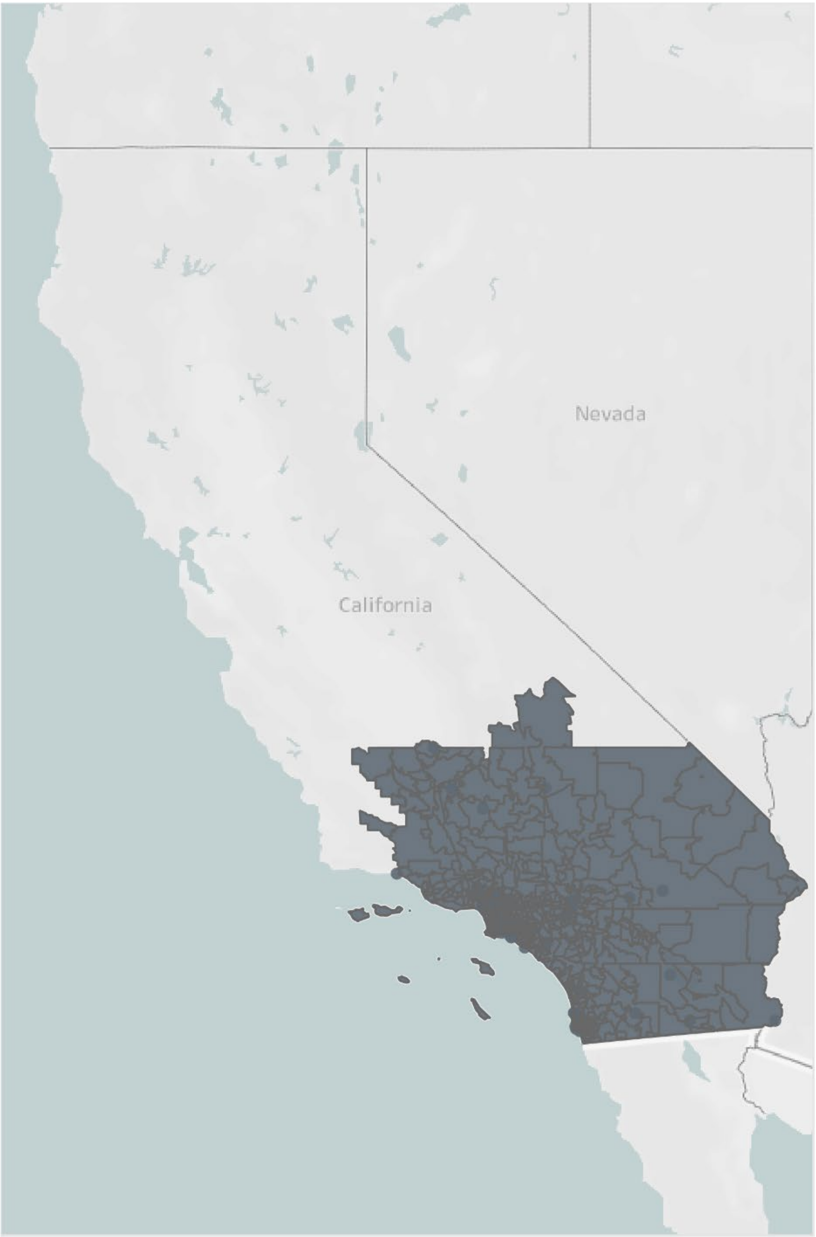

Fig S5. Approximate catchment area for the Kaiser Permanente Southern California Network

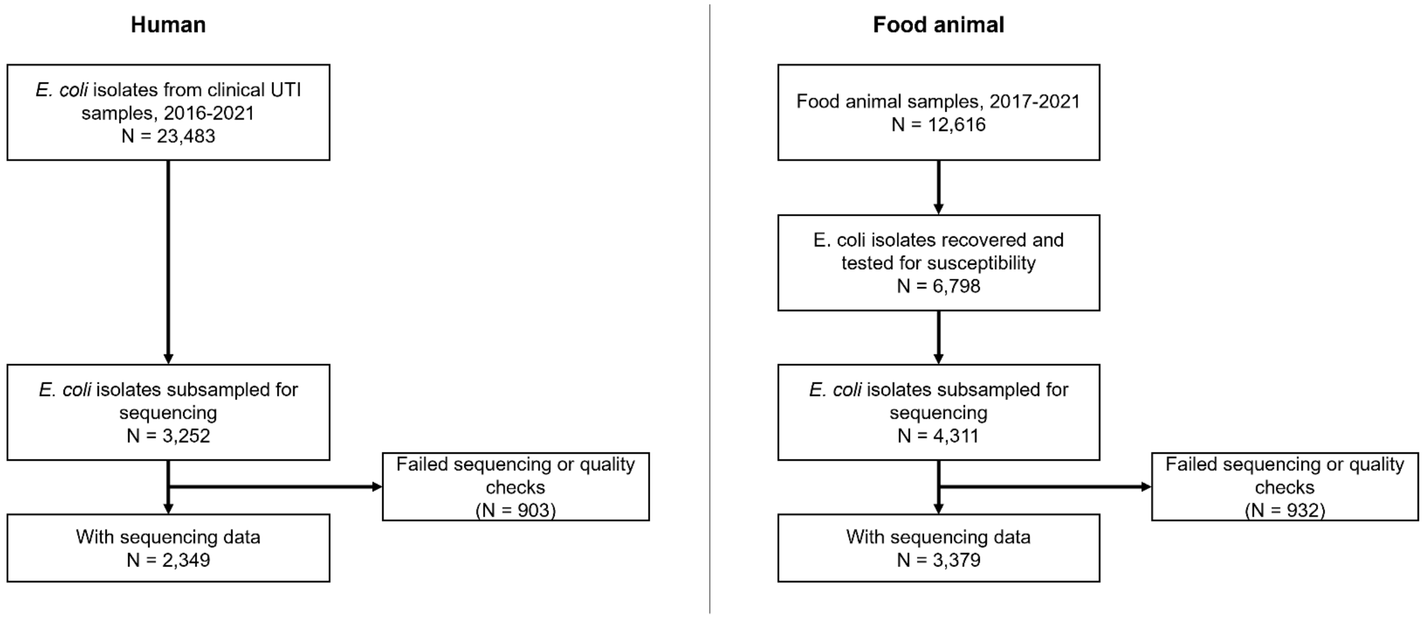

Fig S6. Flowchart of isolate selection and sequencing.

Table S6. Comparing total clinical *E. coli* isolate collection to final sequenced clinical *E. coli* isolate collection.

	**Total clinical**	**Sequenced Clinical**	**p-value**
	**(n=31644)**	**(n=2349)**	
**Sex, n (col %)**			0.020
Female	26924 (85.7)	1723 (87.6)	
Male	4490 (14.3)	244 (12.4)	
**Age, median (Q1-Q3)**	51.9 (31.4-68.8)	53.0 (33.0-69.0)	
Female	50.5 (30.9-68.3)	50.0 (31.0-68.0)	
Male	58.5 (36.7-71.5)	65.5 (54.0-75.5)	
**Body Mass Index, median (Q1-Q3)**	26.9 (23.0-31.5)	27.1 (23.2-31.5)	
Female	26.7 (22.8-31.3)	26.7 (23.0-31.2)	
Male	28.1 (24.4-32.3)	28.6 (25.3-33.0)	
**Race and Ethnicity**			<0.001
Asian	2482 (7.8)	147 (6.3)	
Black	1984 (6.3)	120 (5.1)	
Hispanic	13659 (43.2)	862 (36.7)	
White	11684 (36.9)	738 (31.4)	
Other	1835 (5.8)	482 (20.5)	
**Encounter class, n (col %)**			<0.001
Ambulatory	20024 (63.3)	1322 (56.3)	
Virtual	9334 (29.5)	540 (23.0)	
Other	2285 (7.2)	487 (20.7)	

Table S7. Antimicrobial susceptibility for isolates from meat, zoonotic infections, non-zoonotic infections comparing phenotypic susceptibility to in silico predicted (ResFinder) susceptibility

	% Isolates (n)					
	**ResFinder Susceptibility Predictions**		**Lab Susceptibility Testing Among Sequenced Samples**		**Lab Susceptibility Testing Among All Isolates**	
	**Meat Isolates (n=2559)**	**Human Clinical Isolates (n=2349)**	**Meat Isolates (n=2559)**	**Human Clinical Isolates (n=2349)**	**Meat Isolates (n=4727)**	**Human Clinical Isolates (n=3074)**
**Resistance to Antimicrobial Classes**						
0	54.8 (1402/2559)	51.1 (1200/2349)	36.4 (932/2559)	57.7 (1356/2349)	35.3 (1669/4727)	47.1 (1447/3074)
≥ 1	45.2 (1157/2559)	48.9 (1149/2349)	63.6 (1627/2559)	42.3 (993/2349)	64.7 (3058/4727)	52.9 (1627/3074)
≥ 2	30.1 (769/2559)	37.0 (870/2349)	36.5 (934/2559)	25.1 (589/2349)	38.9 (1837/4727)	31.5 (968/3074)
≥ 3 (multidrug resistant)	17.4 (444/2559)	29.8 (699/2349)	14.5 (371/2559)	12.4 (290/2349)	16.8 (794/4727)	16.0 (493/3074)
**Resistance to Individual Antimicrobials (class)**						
Amoxicillin Clavulanic Acid (β-Lactam/β-Lactamase Inhibitor)[1]	1.3 (34/2559)	2.0 (48/2359)	19.1 (258/2559)	N/A[2]	12.4 (586/4727)	N/A[2]
Ampicillin (β-lactam)[1]	13.2 (337/2559)	38.9 (918/2349)	20.1 (513/2559)	34.5 (811/2349)	20.5 (967/4727)	42.4 (1304/3074)
Azithromycin (Macrolide)	0.2 (6/2559)	13.5 (316/2349)	4.9 (124/2559)	N/A[2]	5.9 (280/4727)	N/A[2]
Cefoxitin (Cephalosporin)	1.1 (29/2559)	0.5 (12/2349)	3.1 (78/2559)	N/A[2]	3.7 (175/4727)	N/A[2]
Chloramphenicol (Amphenicol)	2.0 (51/2559)	3.2 (74/2349)	3.3 (84/2559)	N/A[2]	4.2 (199/4727)	N/A[2]
Ciprofloxacin (Fluoroquinolone)[1]	0.7 (18/2559)	5.1 (120/2349)	2.7 (69/2559)	13.8 (324/2349)	6.1 (287/4727)	15.3 (469/3074)
Colistin (Polymyxin)	0.0 (0/2559)	0.0 (1/2349)	0.0 (0/2559)	N/A[2]	0.0 (0/4727)	N/A[2]
Doxycycline (Tetracycline)	36.5 (933/2559)	24.5 (576/2349)	9.1 (232/2559)	N/A[2]	7.6 (357/4727)	N/A[2]
Gentamicin (Aminoglycoside)	11.7 (300/2559)	6.9 (161/2349)	11.6 (297/2559)	5.3 (124/2349)	10.2 (483/4727)	6.7 (206/3074)
Kanamycin (Aminoglycoside)	8.0 (204/2559)	1.7 (39/2349)	14.8 (378/2559)	N/A[2]	13.9 (656/4727)	N/A[1]

Piperacillin-Tazobactam (β-Lactam/β-Lactamase Inhibitor)[1]	1.3 (34/2559)	2.0 (48/2349)	N/A[2]	N/A[2]	N/A[2]	N/A[2]
Streptomycin (Aminoglycoside)	24.0 (615/2559)	29.7 (698/2349)	44.0 (1127/2559)	N/A[2]	44.7 (2113/4727)	N/A[2]
Sulfamethoxazole (Sulfonamide)	19.4 (497/2559)	29.7 (698/2349)	N/A[2]	N/A[2]	N/A[2]	N/A[2]
Tetracycline	36.5 (933/2559)	24.5 (576/2349)	33.1 (847/2259)	12.3 (288/2349)	34.4 (1627/4727)	17.1 (524/3074)
Trimethoprim (Antifolate)	3.5 (89/2559)	24.3 (570/2349)	N/A[2]	N/A[2]	N/A[2]	N/A[2]
Trimethoprim-Sulfamethoxazole (Antifolate combination)[1]	N/A[3]	N/A[3]	6.0 (153/2559)	19.2 (452/2349)	6.5 (306/4727)	23.8 (731/3074)

[1] In a class of antibiotics commonly used to treat uncomplicated *E. coli* urinary tract infections in the KPSC network, based on Jennifer H Ku, Katia J Bruxvoort, S Bianca Salas, et al. Multidrug Resistance of Escherichia coli From Outpatient Uncomplicated Urinary Tract Infections in a Large United States Integrated Healthcare Organization, *Open Forum Infectious Diseases*, Volume 10, Issue 7, July 2023, ofad287, https://doi.org/10.1093/ofid/ofad287
[2] *N/A[1] (Not Applicable): The antimicrobial drugs were not tested in the lab.

[3] *N/A[2] (Not Applicable): The antimicrobial drugs were tested in the lab but are not listed in the ResFinder database.

Table S8. Characteristics of the 17 source-associated mobile genetic elements (MGEs) identified in (1), including MGE type, number of source-associated accessory genes per element, source association, known features and putative selective functions

MGE	No. genes	Reference genome	Meat or human associated	Possible selective function
H1	3	FMC_2547	Human	Element is three hypothetical proteins, including one with a helix-hairpin-helix DNA binding domain, which might have a role in gene regulation involved in adapting to a human host. In reference genome, genes are in separate operons with other hypothetical proteins. Other adjacent genes include a putative mobile genetic element containing the virulence factor tcpC. (2)
H2	2	FMC_2547	Human	Needs further study. Element is two putative phage tail proteins. In reference genome, these genes are in an operon with many other phage-related proteins, potentially a prophage. It is unclear why the phage tail proteins in particular would be associated with human infections.
H3	20	FMC_2624	Human	Element is all but two genes (clbK and Q) from a colibactin biosynthesis cluster, a well-studied virulence factor for UPEC (3, 4), and three additional nearby genes.
H4	2	FMC_2547	Human	Needs further study. Element comprised of two conserved hypothetical proteins. The only other genes on the reference genome encode a putative MobA family protein (5), replication initiation protein, and a third hypothetical protein.
H5	2	FMC_2547	Human	Element is a putative MbeB mobilization protein (5)and a hypothetical protein. In the reference genome, it is adjacent to putative MbeC and rop/rom genes, which controls plasmid copy number (6)(and therefore copy number of plasmid-borne virulence factors).
H6	11	FMC_1856	Human	Element includes a cluster of membrane and periplasmic proteins, including predicted iron and heme transporters (7, 8), and a scsC-like copper resistance protein (9, 10).
M1	11	FMC_2547	Meat (Pork & Chicken)	Element is a cluster of phage-related genes. In the reference genome, element is interrupted by additional phage-related genes, potentially forming a complete prophage. Element splits the lexA repressor (11) from the DNA damage repair protein recN (12), which may affect the SOS response and DNA damage repair, and is also followed by the toxin- antitoxin pair PasTI (13).
M10	2	FMC_2624	Meat (Turkey)	Element comprised of a LysR-type transcriptional regulator- which may control many infection-related genes (14)- and a sodium-glutamate symporter-glutamate involved in stress responses (15).
M11	6	100_CN_05_B 6_ M2_C4_P2	Meat (Turkey)	Element is a transposable element, adjacent to EL41.

M2	10	57_M1	Meat (Chicken)	Element is a cluster of phage-related genes. In the reference genome, element is interrupted by additional phage-related genes, potentially forming a complete prophage. Element splits xerC and xerD, which may affect gene expression and therefore DNA damage repair (16).
M3	42	FMC_2624	Meat (Poultry)	Element is conjugation machinery (17) - allows spread of potentially beneficial DNA between bacteria. In reference genome, adjacent genes include additional conjugation-related genes.
M4	27	57_M1	Meat (Poultry)	Located on the ColV plasmid (18, 19) – element includes a putative Colicin E1 and immunity protein (20), VapBC (21) and ArtAB (22) toxin-antitoxin proteins (13), multidrug resistance efflux pump mdtH (23, 24), iron and heme transport proteins (18). In the reference genome, element genes are somewhat dispersed on the plasmid.
M5	15	57_M1	Meat (Poultry)	Located on the ColV plasmid (18, 19)– element includes a putative efflux pump (gene homology with MacAB-CusC) (25, 26), a Mig-14-family protein (27), and the colicin V-encoding gene (28). In the reference genome, element genes are somewhat dispersed on the plasmid.
M6	3	57_M1	Meat (Poultry)	Located on the ColBM plasmid (29)– element is a continuous gene cluster comprised of the immunity proteins for colicin M and B and colicin M (28). In the reference genome, element located within a larger toxin-antitoxin gene cluster that includes colicin B (13).
M7	4	57_M1	Meat (Chicken)	Located on the ColBM plasmid (29) – element is a continuous gene cluster comprised of streptomycin and aminoglycoside resistance genes, groEL/groES chaperone (30).
M8	19	100_CN_05_ B6_ M2_C4_P2	Meat (Turkey)	Element includes a copper- and silver-resistance gene cluster (9). In reference genome, intervening genes are additional copper and silver resistance genes.
M9	2	100_CN_05_ B6_ M2_C4_P2	Meat (Turkey)	Element is a pair of transposases. In the reference genome, an additional transposase and recombinase are between the genes, and it is located next to P fimbriae papC (7) and 2 neomycin resistance genes - insertion may affect gene expression.

A. SUPPLEMENTARY REFERENCES

1. Liu CM, Aziz M, Park DE, Wu Z, Stegger M, Li M, Wang Y, Schmidlin K, Johnson TJ, Koch BJ, Hungate BA, Nordstrom L, Gauld L, Weaver B, Rolland D, Statham S, Hall B, Sariya S, Davis GS, Keim PS, Johnson JR, Price LB. 2023. Using source-associated mobile genetic elements to identify zoonotic extraintestinal E. coli infections. One Health 16:100518.

2. Ou Q, Fang J-Q, Zhang Z-S, Chi Z, Fang J, Xu D-Y, Lu K-Z, Qian M-Q, Zhang D-Y, Guo J-P, Gao W, Zhang N-R, Pan J-P. 2021. TcpC inhibits neutrophil extracellular trap formation by enhancing ubiquitination mediated degradation of peptidylarginine deiminase 4. Nat Commun 12:3481.

3. Morgan RN, Saleh SE, Farrag HA, Aboulwafa MM. 2019. Prevalence and pathologic effects of colibactin and cytotoxic necrotizing factor-1 (Cnf 1) in Escherichia coli: experimental and bioinformatics analyses. Gut Pathog 11:22.

4. Faïs T, Delmas J, Barnich N, Bonnet R, Dalmasso G. 2018. Colibactin: More Than a New Bacterial Toxin. Toxins (Basel) 10:151.

5. Francia MV, Varsaki A, Garcillán-Barcia MP, Latorre A, Drainas C, de la Cruz F. 2004. A classification scheme for mobilization regions of bacterial plasmids. FEMS Microbiol Rev 28:79–100.

6. Tomizawa J. 1990. Control of ColE1 plasmid replication. Interaction of Rom protein with an unstable complex formed by RNA I and RNA II. J Mol Biol 212:695–708.

7. Sarowska J, Futoma-Koloch B, Jama-Kmiecik A, Frej-Madrzak M, Ksiazczyk M, Bugla-Ploskonska G, Choroszy-Krol I. 2019. Virulence factors, prevalence and potential transmission of extraintestinal pathogenic Escherichia coli isolated from different sources: recent reports. Gut Pathog 11:10.

8. Subashchandrabose S, Mobley HLT. 2015. Virulence and Fitness Determinants of Uropathogenic Escherichia coli. Microbiol Spectr 3.

9. Cheng G, Ning J, Ahmed S, Huang J, Ullah R, An B, Hao H, Dai M, Huang L, Wang X, Yuan Z. 2019. Selection and dissemination of antimicrobial resistance in Agri-food production. Antimicrob Resist Infect Control 8:158.

10. Shepherd M, Heras B, Achard MES, King GJ, Argente MP, Kurth F, Taylor SL, Howard MJ, King NP, Schembri MA, McEwan AG. 2013. Structural and functional characterization of ScsC, a periplasmic thioredoxin-like protein from Salmonella enterica serovar Typhimurium. Antioxid Redox Signal 19:1494–506.

11. Butala M, Zgur-Bertok D, Busby SJW. 2009. The bacterial LexA transcriptional repressor. Cell Mol Life Sci 66:82–93.

12. Keyamura K, Hishida T. 2019. Topological DNA-binding of structural maintenance of chromosomes-like RecN promotes DNA double-strand break repair in Escherichia coli. Commun Biol 2:413.

13. Norton JP, Mulvey MA. 2012. Toxin-antitoxin systems are important for niche-specific colonization and stress resistance of uropathogenic Escherichia coli. PLoS Pathog 8:e1002954.

14. Maddocks SE, Oyston PCF. 2008. Structure and function of the LysR-type transcriptional regulator (LTTR) family proteins. Microbiology (Reading) 154:3609–3623.

15. Feehily C, Karatzas KAG. 2013. Role of glutamate metabolism in bacterial responses towards acid and other stresses. J Appl Microbiol 114:11–24.

16. Rudenko G, Hohenester E, Muller YA. 2001. LG/LNS domains: multiple functions -- one business end? Trends Biochem Sci 26:363–8.

17. Cabezón E, Ripoll-Rozada J, Peña A, de la Cruz F, Arechaga I. 2015. Towards an integrated model of bacterial conjugation. FEMS Microbiol Rev 39:81–95.

18. Johnson TJ, Siek KE, Johnson SJ, Nolan LK. 2006. DNA sequence of a ColV plasmid and prevalence of selected plasmid-encoded virulence genes among avian Escherichia coli strains. J Bacteriol 188:745–58.

19. Johnson TJ, Logue CM, Wannemuehler Y, Kariyawasam S, Doetkott C, DebRoy C, White DG, Nolan LK. 2009. Examination of the source and extended virulence genotypes of Escherichia coli contaminating retail poultry meat. Foodborne Pathog Dis 6:657–67.

20. Duché D, Issouf M, Lloubès R. 2009. Immunity protein protects colicin E2 from OmpT protease. J Biochem 145:95–101.

21. Winther KS, Gerdes K. 2009. Ectopic production of VapCs from Enterobacteria inhibits translation and trans-activates YoeB mRNA interferase. Mol Microbiol 72:918–30.

22. Littler DR, Ang SY, Moriel DG, Kocan M, Kleifeld O, Johnson MD, Tran MT, Paton AW, Paton JC, Summers RJ, Schembri MA, Rossjohn J, Beddoe T. 2017. Structure-function analyses of a pertussis-like toxin from pathogenic Escherichia coli reveal a distinct mechanism of inhibition of trimeric G-proteins. J Biol Chem 292:15143–15158.

23. Zhang Y, Dong R, Zhang M, Gao H. 2018. Native efflux pumps of Escherichia coli responsible for short and medium chain alcohol. Biochem Eng J 133:149–156.

24. Yu L, Li W, Li Q, Chen X, Ni J, Shang F, Xue T. 2020. Role of LsrR in the regulation of antibiotic sensitivity in avian pathogenic Escherichia coli. Poult Sci 99:3675–3687.

25. Souabni H, Batista Dos Santos W, Cece Q, Catoire LJ, Puvanendran D, Bavro VN, Picard M. 2021. Quantitative real-time analysis of the efflux by the MacAB-TolC tripartite efflux pump clarifies the role of ATP hydrolysis within mechanotransmission mechanism. Commun Biol 4:493.

26. Chacón KN, Mealman TD, McEvoy MM, Blackburn NJ. 2014. Tracking metal ions through a Cu/Ag efflux pump assigns the functional roles of the periplasmic proteins. Proc Natl Acad Sci U S A 111:15373–8.

27. McPhee JB, Small CL, Reid-Yu SA, Brannon JR, Le Moual H, Coombes BK. 2014. Host defense peptide resistance contributes to colonization and maximal intestinal pathology by Crohn's disease-associated adherent-invasive Escherichia coli. Infect Immun 82:3383–93.

28. Cascales E, Buchanan SK, Duché D, Kleanthous C, Lloubès R, Postle K, Riley M, Slatin S, Cavard D. 2007. Colicin biology. Microbiol Mol Biol Rev 71:158–229.

29. Johnson TJ, Johnson SJ, Nolan LK. 2006. Complete DNA sequence of a ColBM plasmid from avian pathogenic Escherichia coli suggests that it evolved from closely related ColV virulence plasmids. J Bacteriol 188:5975–83.

30. Fourie KR, Wilson HL. 2020. Understanding GroEL and DnaK Stress Response Proteins as Antigens for Bacterial Diseases. Vaccines (Basel) 8.
